# Development of Preliminary Criteria of Macrophage Activation Syndrome in Multisystem Inflammatory Syndrome Associated with COVID-19 in Children

**DOI:** 10.3390/biomedicines12122868

**Published:** 2024-12-17

**Authors:** Ilia S. Avrusin, Liudmila V. Bregel, Olesya S. Efremova, Mikhail M. Kostik

**Affiliations:** 1 Hospital Pediatrics, Saint Petersburg State Pediatric Medical University, Saint Petersburg 194100, Russia; avrusin95@gmail.com; 2 Department of Pediatrics, Irkutsk State Medical Academy of Postgraduate Education, A Branch of the Russian Medical Academy of Continuous Professional Education, Irkutsk 664049, Russia; loudmilabregel@yandex.ru; 3 Department of Cardiology, Irkutsk Regional Children’s Clinical Hospital, Irkutsk 664022, Russia; shaguno@mail.ru

**Keywords:** multisystem inflammatory syndrome in children, COVID-19, hemophagocytosis, macrophage activation syndrome

## Abstract

**Background:** Macrophage activation syndrome (MAS) can be regarded as a key factor determining the severity of multisystem inflammatory syndrome associated with COVID-19 in children (MIS-C), and often requires treatment in the intensive care unit (ICU) to avoid life-threatening complications. No reputable specific criteria for the diagnosis of MAS in MIS-C patients have yet been identified, and criteria currently used for the diagnosis of hemophagocytic syndromes, such as HLH-2004, MAS-2005, and MAS-2016, are not sufficient for MAS in MIS-C. Our goal in this study was to work out the criteria for the early diagnosis of MAS in MIS-C. **Methods:** One hundred and sixty-six (166) patients with MIS-C were assessed retrospectively. The two most experienced experts independently identified patients with MAS. The patients were divided into three cohorts: MAS (*n* = 19), without MAS (*n* = 78), and probable MAS (*n* = 67). The latter included patients diagnosed with MAS by only one expert, and it was excluded from the analysis. **Results:** The age of patients with MAS was much higher, and they more frequently had edematous syndrome, hypotension and/or shock, splenomegaly, and CNS involvement. In their blood tests, thrombocytopenia, hypoalbuminemia, and hypertriglyceridemia occurred more often. The level of biomarkers of inflammation, such as ferritin, CRP, troponin, AST, and ALT, was also higher in this group. Increased fibrinogen and D-dimer were also found, demonstrating hypercoagulation in the MAS-MIS-C group. We chose 21 continuous and categorical variables with statistical significance, out of which 2—ferritin > 469 μg/L or platelets < 114 × 10^9^/L—allowed us to discriminate MAS patients. **Conclusions:** Ferritin > 469 μg/L or platelets < 114 × 10^9^/L can be regarded as key signs to differentiate MAS in MIS-C patients with a sensitivity of 100% and specificity of 94.9%, and they can be used along with other diagnostic methods.

## 1. Introduction

Macrophage activation syndrome (MAS) is a severe, life-threatening condition characterized by hemophagocytosis, pancytopenia, coagulopathy, and liver and central nervous system dysfunction [1]. The key signs of MAS were described by A. Ravelli et al. [2]. MAS is more frequently observed in patients with systemic juvenile idiopathic arthritis (sJIA), but can also accompany other rheumatic diseases, including Kawasaki disease (KD) [3,4].

It is known that the COVID-19 pandemic brought new challenges and even possibly a new disease in children, which is multisystem inflammatory syndrome (MIS-C) [5,6]. It has very much in common with KD, as some patients fully or partially meet the American Heart Association (AHA) criteria for KD [7]. However, they also have certain differences [8,9,10,11,12].

The age of children with MIS-C is usually higher, around 6–12 years, but KD patients are much younger, as the majority of them are children under 5 years of age. Moreover, being older than 5 years is considered to be one of the differentiating criteria of MIS-C [12]. Cardiac disorders (myocarditis and pericarditis), often leading to systolic myocardial dysfunction and shock [13,14,15,16,17,18], are also seen more frequently in MIS-C, as well as gastrointestinal symptoms (diarrhea, abdominal pain, and vomiting). Inflammatory markers, thrombocytopenia, hypoalbuminemia, and significant D-dimer elevation are more developed in MIS-C patients and can be important features of severe disease [8,9,10,11,13,16,19,20].

MIS-C is not a frequent, but emergency condition, with a frequency of 1:3000–4000 children who have had COVID-19 [21,22] often treated in an intensive care unit (ICU) (about 50%), as described in several studies [10,12,13,15,17,20,23]. The mortality level of MIS-C is around 1–2% [17,18]. The most important factors determining the severity of the disease are cardiovascular disorders, central nervous system involvement, the respiratory system, and hemodynamic disorders. According to many authors, one of the key factors of MIS-C severity is also MAS. A severe course of MIS-C can result in many signs of it, such as hyperferritinemia and cytopenias [13,20,24,25]. In the literature, signs of hemophagocytic in MIS-C are described much more often than in KD (18.4% vs. 1.2%, respectively) [26].

Currently, there are no reputable specific criteria to assess MAS in MIS-C patients. Thus, multiple studies provide different incidences of it, depending not only on the characteristics of the sample itself but also on the method used to diagnose MAS, ranging from 18% to 76%, according to data from different authors [9,13,24,26,27,28].

Unfortunately, these criteria are not optimal for evaluating MAS in MIS-C. This might be why the range of reported MAS incidence in patients with MIS-C is so wide.

This study aimed at identifying specific criteria for early diagnosis of MAS in MIS-C patients.

## 2. Materials and Methods

### 2.1. Study Design

The retrospective multi-center cohort study included data from 166 patients (99 boys, 67 girls), aged 4 months to 17 years (median 8.2 years), who were hospitalized with the diagnosis of “multisystem inflammatory syndrome associated with COVID-19 in children” to the clinics of Saint Petersburg, Irkutsk, and other cities of Russia during the pandemic (from June 2020 to June 2022). All patients met the WHO criteria of MIS-C [6].

### 2.2. COVID-19 Confirmation

COVID-19 in anamnesis was confirmed as follows:

(1) Positive PCR with reverse transcription (13%);
(2) Identified antibodies to SARS-CoV-2 of classes Ig M (40.3%) or Ig G (97.4%);
(3) Close contact with a person with confirmed COVID-19 (65.6%).

### 2.3. Assessments

We assessed the occurrence of a total of 80 clinical signs and laboratory abnormalities in patients with MIS-C.

(i) Clinical symptoms: signs of abdominal disorders (stomach ache, diarrhea, and vomiting), respiratory system disorders, and nervous system disorders, as well as such symptoms as fever and its duration, rash, conjunctivitis, xerochilia, lymphadenopathy, swelling/reddening of hands/feet, sore throat, hyperemia of mucous membranes, skin exfoliation on fingers, edema, hepatomegaly, splenomegaly, and low blood pressure/shock were taken into account.

(ii) Laboratory parameters: the levels of erythrocytes (RBC), hemoglobin, leukocytes (WBC), platelets (PLT), erythrocyte sedimentation rate (ESR), C-reactive protein (CRP), ferritin, alanine aminotransferase (ALT), aspartate aminotransferase (AST), serum protein, albumin, triglycerides, creatinine, lactate dehydrogenase (LDH), and troponin, as well as blood coagulation parameters such as fibrinogen and D-dimer were included in the assessments.

(iii) Instrumental data: heart involvement was evaluated based on echocardiography and electocardiography data.

(iv) Treatment: glucocorticosteroids, acetylsalicylic acid, and intravenous immunoglobulin biological therapy (tocilizumab) and supportive care treatment.

(v) MAS detection: Signs of hemophagocytosis were determined by assessing patient compliance with known criteria for these conditions (HLH-2004 [29], MAS 2005 [30], and MAS 2016 [31]), as well as calculating the Hscore [32]. The highest level of abnormalities was considered. Two key opinion leaders (LVB and MMK) selected MAS cases independently in a blinded manner. If both experts were in agreement, the patients were included in the MAS group (*n* = 19) and no MAS (*n* = 78) groups. Cases on which the experts discorded (patients with probable MAS, *n* = 67) were excluded from the analysis. As a next step, a comparative analysis of the two remaining groups with and without MAS was conducted.

### 2.4. Statistics

We utilized the STATISTICA software package, version 10.0 (StatSoft Inc., St. Tulsa, OK, USA). Numerical indicators were presented with the median (25th; 75th percentiles), and categorical variables were presented with absolute number and the parts (%). We used the Pearson criteria χ^2^ for the comparison of the independent categorical variables and the Mann–Whitney criteria for independent numerical variables. The ability of each predictor to discriminate MAS patients was assessed with an analysis of the sensitivity and specificity. The cut-off values of the quantitative variables were calculated with AUC-ROC analysis (AUC—area under the curve) with the determination of 95% confidence interval (CI) and calculation of the odds ratio (OR) without taking into account the time of development of events of interest using 2 × 2 tables. Sensitivity (Se) and specificity (Sp) were calculated for each significant predictor. Independent predictors were evaluated with multiple regression analysis. We selected only clinically meaningful parameters with the highest sensitivity, specificity, and OR and excluded duplications and overlapped parameters from the set of significant tests, obtained after the univariate analysis for the following multiple regression analysis. Differences were considered statistically significant if the *p*-value was less than 0.05.

## 3. Results

### 3.1. Characteristics of Patients with MIS-C

One hundred and sixty-six (166) patients with MIS-C were assessed. There was a slight prevalence of male patients (59.6%) in the studied group. The patients were 4 months–17 years old, with a median of 8 years, 2 months. Patients were treated in the hospital for 18 days on average. No patients died in the studied cohort. All patients fully recovered.

The patients mostly had a fever (100%), conjunctivitis (84.8%), rash (78.9%), abdominal disorders (77.2%) such as nausea, stomach ache, vomiting, and diarrhea, lymphadenopathy (66.9%), hyperemia of mucous (64%), hepatomegaly (64.4%), splenomegaly (40.7%), hands and feet reddening or edema (62.4%), sore throat (56.3%), oedematous syndrome (50.5%), pulmonary disorders (49.4%), xerochilia (49.3%), neurological symptoms (47.8%), and low blood pressure/shock (43.8%).

Many patients had signs of hemophagocytosis: ferritin (n = 69/90, 76.7%); high levels of liver enzymes: ALT (n = 85/164, 52.8%), AST (n = 101/148, 68.2%), LDH (n = 56/94, 59.6%), D-dimer (n = 120/125, 96.0%), hypoalbuminemia (n = 115/182, 81.0%), and hypoproteinemia (n = 102/126, 81.0%); and signs of inflammation—elevation of ESR (n = 150, 90.9%) and CRP (n = 153/157, 97.5%).

Almost one-third of patients had signs of myocardial damage (30.6%) and pericarditis (28.8%); some patients also had coronary artery dilatation/aneurysms (15.8%).

Patients were usually treated with glucocorticosteroids (81.5%); some of them also received acetylsalicylic acid (57.1%) and immunoglobulin infusions (44.7%, due to restricted access), and 4.9% of patients required biological therapy (tocilizumab). As supportive care, albumin, plasma, and erythrocyte mass were infused, and inotropic therapy was used.

### 3.2. Comparison of MAS and No MAS Groups

The age of children in the MAS group was much higher with a median age of 9 years and 8 months, compared to patients without MAS with a median age of 6 years and 4 months (*p* = 0.007). There were proportionally more boys in both cohorts.

Patients with MAS had sore throats more frequently (88.2% vs. 52.7%, *p* = 0.027), as well as edema (86.7% vs. 31.6%, *p* = 0.0006) and neurological disorders, from headache to varying degrees of depression of consciousness from somnolence to coma (83.3% vs. 40.8%, *p* = 0.005). Patients with MAS had hepatomegaly (88.2% vs. 56.7%, *p* = 0.055) and splenomegaly (94.1% vs. 26.9%, *p* = 0.000003) much more often. Shock/hypotension was found in MAS patients almost three times more often than in patients without MAS (68.4% vs. 23.1%, *p* = 0.0001).

Heart involvement was represented by myocardial involvement (77.8% vs. 26.3%, *p* = 0.00004) and pericarditis (66.7% vs. 22.4%, *p* = 0.0003) in patients with MAS significantly more often, while coronary artery dilation (0% vs. 26.7%, *p* = 0.016) was more typical for patients without MAS, and was not found in any MAS patient.

There were significant differences in laboratory parameters specific to MAS. Thus, 100% of patients from the MAS group had increased ferritin levels (1276.6 μg/L), while in patients without MAS, only 63.4% had hyperferritinemia with a median level of 194.1 μg/L (*p* = 0.0000001). Also, patients with MAS had signs of cytopenia much more frequently: median hemoglobin level was 98 g/L vs. 109 g/L (*p* = 0.009); MAS patients had thrombocytopenia in 94.7% of cases, while patients without MAS had it only in 18% (*p* = 0.0000001), with a median level of PLT 71 × 10^9^/L, vs. 444 × 10^9^/L (*p* = 0.0000001). In addition, patients with MAS had higher levels of ALT (62 IU/L vs. 30.1 IU/L, *p* = 0.0003), AST (68.7 vs. 38.0 IU/L, *p* = 0.00001), and triglycerides (3.6 mmol/L vs. 2.0 mmol/L, *p* = 0.006), and, as expected, they had lower median fibrinogen levels (2.7 g/L vs. 5.5 g/L, *p* = 0.0000001).

Moreover, in the MAS group, higher levels of CRP (23.2 mg/dL vs. 9.9 mg/dL, *p* = 0.0006) and D-dimer (3460 ng/mL vs. 944 ng/mL, *p* = 0.0002) were observed.

The presence of MAS/hemofagocytosis was also evaluated with the existing criteria. Almost every patient with MAS fulfilled the 2005 criteria for macrophage activation syndrome [30] (94.7% vs. 4.1%, *p* = 0.0000001), and 68.4% met the MAS 2016 criteria, while in the second group, it was only 1.4% (*p* = 0.0000001). The HLH-2004 criteria are not so convenient for MAS detection, but 42.1% of MAS patients fulfilled them. Additionally, HScore was calculated for both groups, and the median was 165 vs. 75 points for MAS and no MAS patients, respectively (*p* = 0.0000001).

Since the condition and disease progression differed in both groups, there were some differences in treatment approaches. Thus, MAS patients received corticosteroids in 100% of cases, while patients without MAS had it in 72% (*p* = 0.033). Also, MAS patients received biological treatment in 30.8% of cases vs. 1.8% in the second group (*p* = 0.001). The differentials of both cohorts are presented in Table 1.

As the next phase of the analysis, we took statistically significant continuous and categorical variables and analyzed sensitivity and specificity with OR calculation. The results are demonstrated in Table 2.

After that, we chose the most clinically meaningful parameters with the highest sensitivity, specificity, and OR, excluding duplications, e.g., we did not include in the analysis Hscore, correspondence to HLH-2004 criteria, correspondence to MAS 2005 criteria, or correspondence to MAS 2016 criteria because there are duplicates of each other and overlapped with another test, such as fibrinogen, platelets, WBC, triglycerides, ferritin, etc. From the overlapped pair criteria—serum protein ≤ 53.4 g/L and albumin ≤ 26 g/L—we used the first one because it had a higher sensitivity, specificity, and OR, and the *p*-value was more significant. From the overlapped pair criteria, ALT > 45 U/L and AST > 60 U/L were overlapped, but again we used the latter due to a higher sensitivity, specificity, and OR, and more significant p-level.

**Figure 1:**
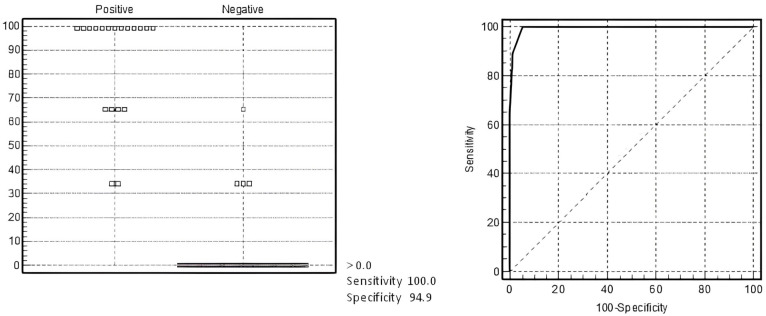
Receiver operating characteristic (ROC) curve analysis for diagnosis of MAS in MIS-C patients with our criteria computed with the developmental dataset. The optimal cutoff was selected as the threshold giving the highest value for the sum of sensitivity and specificity. Area under the curve (AUC) = 0.995 (0.953–0.996); DS for MAS in MIS-C with sensitivity of 94.9% and specificity of 100%.

Thus, multivariate analysis educed two values: ferritin > 469 ng/mL and PLT ≤ 114 × 10^9^/L (Table 3). The best possible cutoff value is identified as a threshold providing the highest level for the total sensitivity and specificity. The area under the curve (AUC) = 0.995 (0.953–0.996), and r^2^ for the whole model = 0.89. Suggested criteria allowed for determining the presence of MAS in MIS-C patients with a sensitivity of 100% and specificity of 94.9% (Figure 1).

## 4. Discussion

The study included 166 patients diagnosed with MIS-C. Then, two opinion leaders in pediatric rheumatology independently chose patients with MAS. After excluding patients about whom expert opinions differed, the remaining patients were subjected to analysis, which resulted in determining specific criteria for defining MAS in patients with MIS-C.

Many manuscripts describe MAS/hemophagocytic syndrome in patients with MIS-C, with an approximate frequency of 18–76% [9,24,26,27,28]. However, for the evaluation of MAS in MIS-C, there have been no specific criteria. That is why the frequency of MAS diagnosis accompanying MIS-C is so variable within relevant studies. For example, in one of the first manuscripts about MIS-C, the presence of MAS in patients was assessed using MAS 2016 criteria [31] and amounted to 50%. However, the sample size was relatively small, so 5 out of 10 patients met the criteria. In the other study, the occurrence of secondary Hemophagocytic Lymphohistiocytosis (sHLH) amounted to 18.4% of children complying with HLH-2004 criteria [26].

In the study by Reiff D.D. et al., the incidence of cytokine storm syndrome (CSS) in patients with MIS-C and active COVID-19 was evaluated. It was evaluated based on the grading approach implemented in MAS and HLH assessments. For example, only 5.6% of patients with severe MIS-C met the HLH-2004 criteria, while 33.3% of patients had MAS according to the MAS 2016 criteria [27]. They also used the COVID-19 CSS Quick Score [33], based on which 70.6% of severe MIS-C patients had cytokine storm syndrome. In addition, the authors used HScore for the evaluation of hemophagocytic syndrome, and the median value in MIS-C patients was 68 [27].

In our study, some of these grading tools were also used. As a result, in our MAS group, 42.1% of patients fulfilled the HLH-2004 [29] criteria, and 68.4% met the criteria for MAS [31]. The median HScore in our study was 91 for the whole group, and for MAS and no MAS groups, it was 165 and 75, respectively (*p* = 0.0000001). In our previous studies, we mentioned that an HScore of 91 was due to the severity of MIS-C and hemophagocytosis [20,34].

The age of children with MAS was higher (median age—9 years and 8 months vs. 6 years and 4 months, *p* = 0.007) than in the non-MAS group.

Regarding clinical signs, in our study, children with MAS frequently had such symptoms of hemophagocytosis as hepatomegaly (88.2%) and splenomegaly (94.1%). They also had shock/hypotension much more often than patients without MAS (68.4% vs. 23.1%, *p* = 0.0001), as well as sore throat (88.2% vs. 52.7%, *p* = 0.027), edema (86.7% vs. 31.6%, *p* = 0.0006), and neurological disorders (83.3% vs. 40.8%, *p* = 0.005).

A similar pattern can be found in the literature. Some authors mention that MIS-C patients with MAS were older (9.0 vs. 7.9 years, *p* = 0.022) and had a higher frequency of lymphadenopathy, hepatomegaly, and splenomegaly [35]. In the study by Lucioni et al., myocardial dysfunction (40% vs. 19%, *p* < 0.0001) was observed more frequently in MIS-C patients with MAS at disease onset [35]. In our study, myocardial damage was observed in 77.8% of the MAS group and 26.3% of the non-MAS patients (*p* = 0.00004).

Similar results are shown in the study by P. Buda et al., where MIS-C patients with MAS had a higher age than other groups (median age was 11.2 and 8.1 years, respectively), and they more often had swelling/reddening of hands and feet (75.5 vs. 49.8%) [28].

Regarding laboratory changes, according to Buda et al., median lymphocyte (0.74 vs. 1.16 × 10^3^/μL) and platelet (140 vs. 188 × 10^3^/μL) counts, albumin (3.1 vs. 3.4 g/dL), and sodium (132 vs. 135 mmol/L) levels were much lower in MIS-C patients with MAS than in patients in the non-MAS group. Similar to the abovementioned tests, the levels of CRP (189.08 vs. 129 mg/L), ferritin (920.13 vs. 292.3 μg/L), D-dimer (3.78 vs. 2.4 mg/L), and triglycerides (210 vs. 140 mg/dL) [28] were much higher in patients with MAS. The same pattern was demonstrated in our study: ferritin (1276.6 μg/L vs. 194.1 μg/L, *p* = 0.0000001), C-RP (23.2 mg/dL vs. 9.9 mg/dL, *p* = 0.0006), triglycerides (3.6 mmol/L vs. 2.0 mmol/L, *p* = 0.006), and D-dimer (3460 ng/mL vs. 944 ng/mL, *p* = 0.0002) were also higher in the MAS group.

Similar results were shown in the study by Lucioni et al., as at MAS onset, patients had higher levels of ferritin (1446 vs. 403 ng/mL, *p* < 0.0001), triglycerides (235 vs. 186 mg/dL, *p* < 0.0001), and AST (60 vs. 35 U/L, *p* < 0.0001), and lower fibrinogen (463 vs. 543 mg/dL, *p* = 0.004) and PLT (133 vs. 193 × 10^9^/L, *p* < 0.0001) levels compared to MIS-C patients without MAS.

In our study, patients had the same pattern of lower levels of fibrinogen (2.7 g/L vs. 5.5 g/L, *p* = 0.0000001) and PLT (71 × 10^9^/L, vs. 444 × 10^9^/L, *p* = 0.0000001) in the MAS group.

As for therapeutic approaches, in our study, corticosteroid (100% vs. 72%, *p* = 0.033) and biological (90.8% vs. 1.8%, *p* = 0.001) treatment was more frequently used in MAS patients. In addition, patients with MAS were admitted to ICU more than twice as frequently compared to patients without MAS (84.2% vs. 30.8%, *p* = 0.00002).

A similar trend was observed in the study by Buda et al.: MIS-C patients with MAS received IVIG more frequently than children without MAS (94.8 vs. 87.6%), as well as corticosteroids (87.3 vs. 62.7%), and they were admitted to ICU twice as often.

Also, in the study by Lucioni et al., patients with MAS were treated with corticosteroids (99% vs. 80%) and needed biologics (Anakinra) (40% vs. 9%) more often than MIS-C patients without MAS [35].

In our research, MAS patients received biological treatment much more often as well (30.8% vs. 1.8%, *p* = 0.001). However, we used tocilizumab instead of anakinra, based on the availability of the drug.

The median duration of hospitalization was higher in MAS patients (25 days vs. 15 days, *p* = 0.026) in our study, corresponding to the observations in Buda et al.’s research (20 days vs. 12 days) [28].

Such a high frequency of corticosteroid use in our patients is due to Russian national recommendations, according to which glucocorticosteroids are considered the treatment of choice [36], while ACR guidelines [37] and British national consensus [38] specify IVIG as the first option, as in Kawasaki disease [7].

The main goal of the study by F. Lucioni et al. was to evaluate the performance of the 2016 classification criteria in recognizing MAS in patients with MIS-C. So, they showed a sensitivity of 80% and a specificity of 83% in recognizing MAS in MIS-C patients [35]. We also evaluated the sensitivity and specificity of some existing criteria, including MAS-2016. The results were as follows: a sensitivity of 42.1% and a specificity of 100.0% for HLH-2004, a sensitivity of 94.7% and a specificity of 95.9% for the MAS-2005 criteria, and a sensitivity of 72.2% and a specificity of 98.6% for the MAS-2016 criteria (EULAR/ACR/PRES). Our criteria showed a sensitivity of 94.9% and a specificity of 100%.

There is currently no consensus regarding hemophagocytic syndrome in MIS-C. Clinically, it can be related to MAS rather than to primary HLH. Since in MIS-C patients, signs of systemic inflammation are more pronounced than in patients with primary HLH, CRP is usually not significantly increased [39,40].

MAS and HLH are not separate entities. They are both characterized by a series of hemophagocytic processes, which are triggered by a failure in cytotoxicity, resulting in a cytokine storm [41], but there are some differences and similarities in the cytokine profile.

At the core of MIS-C may be an immune imbalance causing an abnormal immune reaction to the SARS-CoV-2 virus, resulting in an immense blow-out of cytokines such as interleukin-1β (IL)-1β, IL-6, IL-8, IL-10, and IL-18 [42,43].

Thus, interferon-gamma is the main cytokine in primary HLH, while IL-1, 6, and 17 are predominant in MIS-C [42,43,44,45].

The mechanism of MAS development is associated with the compulsive emission of T-lymphocytes and macrophages against the background of a decrease in the cytotoxic activity of NK cells and T-lymphocytes due to mutations in the genes that control the cytolytic function [46,47]. Cytokines are also key actors in MAS development. Excessive production of interferon γ, associated with constant activation of TLR-9, is most important. It occurs in the presence of a mutation in the IFR5 (interferon regulatory factor 5) gene and has been described in patients with SJIA who developed MAS. Several studies have shown the role of other cytokines: IL1, 4, 6, 12, 16, 18, tumor necrosis factor (TNF) α, sCD25, sCD163, and s100 [48,49,50].

Thus, we can see that MIS-C has a lot in common with MAS, also in the field of cytokine profile. Moreover, treatment options used in MAS-sJIA were highly beneficial in MIS-C.

In addition, there are rare heterozygous missense mutations in genes responsible for primary HLH (LYST, STXBP2, PRF1, UNC13D, AP3B1, and DOCK8) found in patients with MIS-C [51].

It is worth mentioning the study by A. Reiter et al. “Proteomic mapping identify serum marker signatures associated with MIS-C specific hyperinflammation and cardiovascular manifestation”. The authors showed that overexpression of IL-17A in MIS-C and KD was the best marker for the differentiation of this syndrome from hyperinflammation (including MAS). High production of adenosine deaminase and IL-18 were attributes for them. Cardiovascular disorders and myocarditis in MIS-C are related to the reduction in serum TNF-related subfamily member 9 (TNFRSF9) and apoptosis-inducing ligand (TRAIL) [52].

Vaccination against the SARS-CoV-2 virus prevented the disease from spreading and severity in adults [53,54].

Also, it is important to mention that a second dose of the vaccine after 1 month increases the efficacy of the vaccination even against the Omicron strain [55]. So, it seems to be important to carry out timely, accessible, and sufficient education of the population about the problem of COVID-19 prevention. The greatest influence on people’s willingness to be re-vaccinated is exerted by such factors as the level of public knowledge about the problem, as well as stricter compliance with government measures to prevent and control COVID-19 [56].

Theoretically, vaccination may prevent the frequency and severity of MIS-C, and possibly reduce frequency and severity of MAS’s course in pediatric patients. However, there are no evident data in pediatric studies. At the time of broad use of the vaccine, the Delta strain, responsible for the majority of MIS-C cases, was replaced with Omicron and its variants that have a lower ability to provoke MIS-C. On the other hand, there are some reports that vaccines can provoke MIS-C in uninfected children, even with fatal outcomes [57,58].

Hemophagocytosis/macrophage activation syndrome is quite a common and highly severe syndrome in MIS-C, which was demonstrated in many studies [26,27,28].

Unfortunately, no valid diagnostic criteria for hemophagocytosis/MAS in MIS-C currently exist. Many investigators used not fully relevant ones applicable to other diseases, which makes the accuracy of the results questionable [29,30,31].

Although several tools for assessing MAS already exist and the HScore has confirmed its reliability in assessing hemophagocytic syndrome in patients with MIS-C, there was a clear need to develop a specific diagnostic system for assessing hemophagocytosis in patients with MIS-C [27,28,32,34]. The suggested criteria also need to be validated based on bigger cohorts, so there is room for international cooperation.

## 5. Limitations

Our study has several limitations, related to the retrospective type, missing data, and selection into groups, based on the personal opinion of the physicians. The absence of unique criteria and assessments, as well as different times of assessment, leads to some bias, affects the generalizability and repeatability of the results, and decreases the strength of the study. To partially avoid the bias, we used blind separate selection made by the two most experienced physicians.

## 6. Conclusions

The elaborated diagnostic criteria—ferritin > 469 ng/L and platelets <114 × 109/L—allow for the differentiation of MAS in MIS-C patients with a sensitivity of 100% and a specificity of 94.9% and can be a beneficial addition to the other diagnostic methods. Future studies will aim to include a bigger sample size, validation analysis, and detailed assessment of the patients with probable MAS, as well as a comparison of our results with MAS patients of similar inflammatory phenotype, e.g., systemic arthritis.

## Figures and Tables

**Table 1:** Comparison of parameters in MAS and no MAS groups of patients with MIS-C.

Parameter	MAS (*n* = 19)	No MAS (*n* = 78)	*p*-Value
Age, months, Me (25%; 75%)	116 (104; 137)	76 (36; 120)	0.007
Gender, male, n (%)	13 (68.4)	46 (59)	0.449
Clinical parameters			
GI symptoms, n (%)	18 (94.7)	54/75 (71.1)	0.097
Neurological symptoms, n (%)	15/18 (83.3)	31/75 (40.8)	0.005
Sore throat, n (%)	15/17 (88.2)	39/73 (52.7)	0.027
Rash, n (%)	13/18 (72.2)	59/72 (80.8)	0.575
Conjunctivitis, n (%)	14/16 (87.5)	58/70 (81.7)	0.804
Dry cracked lips, n (%)	8/14 (57.1)	28/68 (40.6)	0.492
Bright mucous, n (%)	12/14 (85.7)	34/59 (56.7)	0.128
Respiratory signs, n (%)	13 (68.4)	39/76 (50.7)	0.357
Cervical lymphadenopathy, n (%)	13/15 (86.7)	42/72 (57.5)	0.104
Hands/feet erythema/edema, n (%)	12/16 (75)	34/66 (50.8)	0.207
Peeling of fingers, n (%)	6/13 (46.2)	20/64 (30.8)	0.526
Edematous syndrome, %	13/15 (86.7)	18/56 (31.6)	0.0006
Hepatomegaly, n (%)	15/17 (88.2)	38/66 (56.7)	0.055
Splenomegaly, n (%)	16/17 (94.1)	18/66 (26.9)	0.000003
Shock/hypotension, n (%)	13 (68.4)	18 (23.1)	0.0001
Duration of fever, days	9 (6; 11)	9 (7; 13)	0.844
KD criteria fulfillment, complete, n (%) incomplete, n (%)	8 (42.1) 4 (21.1)	29 (37.2) 19 (24.4)	0.692 0.761
Laboratory parameters			
Erythrocytes, 10^12^/L, Me (25%; 75%)	3.7 (3.2; 3.9)	3.9 (3.7; 4.4)	0.016
Hemoglobin, g/L, Me (25%; 75%)	98 (83; 108)	109 (100; 115)	0.009
White blood cells, 10^9^/L, Me (25%; 75%)	18.6 (13.1; 21.4)	15.9 (12.9; 20.2)	0.484
Platelets, 10^9^/L, Me (25%; 75%)	71 (51; 89)	444 (202; 755)	0.0000001
Thrombocytopenia, n (%)	18 (94.7)	14 (18)	0.0000001
Thrombocytosis, n (%)	0 (0)	42 (53.9)	0.00002
ESR, mm/h, Me (25%; 75%)	40 (27; 45)	41 (27; 54)	0.288
C-reactive protein, mg/dL, Me (25%; 75%)	23.2 (15.3; 24.5)	9.9 (3.0; 20.1)	0.0006
Ferritin, μg/L, Me (25%; 75%)	1276.6 (801.1; 1689.8)	194.1 (88.4; 312.0)	0.0000001
Hyperferritinemia, n (%)	15/15 (100)	26/41 (63.4)	0.006
ALT, IU/L, Me (25%; 75%)	62.0 (43.0; 120.0)	30.1 (18.1; 46.4)	0.0003
Increased ALT, n (%)	15 (79)	25 (32.1)	0.0002
AST, IU/L, Me (25%; 75%)	68.7 (61.0; 164.6)	38.0 (26.0; 53.6)	0.00001
Increased AST, n (%)	17 (89.5)	33/67 (49.3)	0.002
Bilirubin, mcmol/L, Me (25%; 75%)	14.1 (8.3; 26.9)	12.0 (6.4; 16.8)	0.228
Serum protein, g/L, Me (25%; 75%)	45.5 (44.0; 49.0)	59.5 (55.1; 63.9)	0.0000001
Albumin, g/L, Me (25%; 75%)	25.0 (22.0; 28.0)	30.2 (26.7; 34.0)	0.0003
Triglycerides, mmol/L, Me (25%; 75%)	3.6 (2.4; 3.9)	2.0 (1.5; 2.5)	0.006
Increased triglycerides, n (%)	12/19 (63.2)	9/10 (90)	0.124
Creatinin, mmol/L, Me (25%; 75%)	63.6 (54.0; 110.0)	52.1 (39.2; 60.5)	0.005
Increased creatinine, n (%)	7/17 (41.2)	7/58 (12.1)	0.007
LDH, IU/L, Me (25%; 75%)	636.9 (431.0; 800.0)	448.2 (245.7; 644.0)	0.177
Increased LDH, n (%)	11/14 (78.6)	22/44 (50)	0.060
LDH/Ferritin, Me (25%; 75%)	0.4 (0.2; 0.7)	1.7 (0.9; 4.0)	0.049
Fibrinogen, g/L, Me (25%; 75%)	2.7 (1.1; 5.8)	5.5 (4.0; 7.6)	0.0000001
D-dimer, ng/mL, Me (25%; 75%)	3460 (1890; 5569)	944 (586; 1948)	0.0002
Troponin, pg/mL, Me (25%; 75%)	117.7 (14.0; 256.5)	2.0 (0.2; 4.2)	0.079
Hemophagocytosis evaluation			
Hscore	165 (106; 204)	75 (56; 91)	0.0000001
HLH-2004, n (%)	8 (42.1)	0 (0)	0.0000001
MAS 2005, n (%)	18 (94.7)	3/73 (4.1)	0.0000001
MAS 2016, n (%)	13 (68.4)	1/73 (1.4)	0.0000001
Echo findings			
Coronary dilatations/aneurysms, n (%)	0/17 (0)	20/75 (26.7)	0.016
Myocardial damage, n (%)	14/18 (77.8)	20/76 (26.3)	0.00004
Pericardial effusion, n (%)	12/18 (66.7)	17/76 (22.4)	0.0003
Treatment and outcomes			
IVIG treatment, n (%)	10/18 (55.6)	24/75 (32)	0.062
Acetylsalicylic acid, n (%)	7/18 (38.9)	44/68 (63.8)	0.121
Corticosteroids, n (%)	19 (100)	54/74 (72)	0.033
Biologics, n (%)	4/13 (30.8)	1/56 (1.8)	0.001
ICU admission, n (%)	16 (84.2)	24 (30.8)	0.00002
Stay in hospital, for days	25 (17; 31)	15 (11; 21)	0.026

Abbreviations: Me—median; GI—gastrointestinal; KD—Kawasaki disease; ESR—erythrocyte sedimentation rate; LDH—lactate dehydrogenase; ALT—alanine aminotransferase; AST—aspartate aminotransferase; LDH—lactate dehydrogenase; HLH—hemophagocytic lymphohistiocytosis; MAS—macrophage activation syndrome; IVIG—intravenous immunoglobulin; ICU—intensive care unit.

**Table 2:** Parameters associated with MAS in MIS-C.

Parameter	Se	Sp	OR (95%CI)	RR (95%CI)	*p*-Value
Age > 89 months	84.2	58.7	7.6 (2.0; 28.2)	5.3 01.7; 17.1)	0.0008
CNS involvement	83.3	58.7	7.1 (1.9; 26.6)	5.1 (1.6; 16.5)	0.005
Sore throat	88.2	53.0	8.5 (1.8; 39.4)	6.4 (1.5; 26.5)	0.027
Face swelling	72.2	67.9	5.5 (1.7; 17.8)	3.6 (1.4; 9.1)	0.0006
Splenomegaly	94.1	72.7	42.7 (5.3; 345.5)	23.1 (3.2; 165.7)	0.000003
Shock/hypotension	68.4	76.9	7.2 (2.4; 21.7)	4.6 (1.9; 11.0)	0.0001
Hemoglobin ≤ 92 g/L	42.1	91.7	7.3 (2.2; 24.1)	4.3 (2.1; 8.8)	0.0004
Platelets ≤ 114 × 10^9^/L	89.5	98.7	654.5 (56.1; 7640.1)	37.3 (9.5; 36.3)	0.000001
C-reactive protein > 119 mg/L	94.7	56.2	32.1 (2.9; 182.1)	15.1 (2.1; 108.6)	0.00007
Ferritin > 469 ng/mL	93.8	84.2	235.0 (22.7; 2431.5)	14.2 (2.1; 96.0)	0.0000001
LDH/Ferritin ≤ 0.7	80.0	80.0	16.0 (2.4; 106.7)	6.0 (1.5; 23.5)	0.002
ALT > 45 U/L	73.7	73.1	7.6 (2.4; 23.7)	5.0 (2.0; 16.2)	0.0001
AST > 60 U/L	78.9	83.1	18.5 (5.3; 64.7)	9.1 (3.3; 25.1)	0.0000001
Serum protein ≤ 53.4 g/L	94.4	84.6	93.5 (11.2; 784.0)	35.3 (5.0; 251.3)	0.0000001
Albumin ≤ 26 g/L	58.8	80.0	5.7 (1.9; 17.7)	3.8 (1.6; 8.7)	0.001
Triglycerides > 2.4 mmol/L	81.8	72.7	12.0 (2.0; 72.4)	5.4 (1.4; 21.3)	0.003
Creatinine > 62.5 mcmol/L	55.6	81.3	5.5 (1.8; 16.3)	3.6 91.6; 8.0)	0.001
Fibrinogen < 2.4 g/L	50.0	96.9	25.2 (4.7; 136.2)	5.8 (3.0; 11.2)	0.000002
D-dimer > 2270 ng/mL	70.6	85.2	13.8 (3.8; 49.9)	6.1 (2.5; 15.2)	0.000008
Troponin > 5.3 pg/m	87.5	81.3	30.3 (2.6; 348.9)	9.8 (1.4; 67.6)	0.001
Hscore > 113 U	73.7	88.7	22.1 (8.3; 77.6)	8.7 (3.5; 21.3)	0.0000001
Correspondence to HLH-2004 criteria	42.1	100.0	-	8.1 (4.7; 14.1)	0.0000001
Correspondence to MAS 2005 criteria	94.7	95.9	426.0 (41.8; 4341.6)	61.7 (8.8; 435.6)	0.0000001
Correspondence to MAS 2016 criteria	72.2	98.6	187.2 (20.2; 1735.4)	14.3 (6.1; 33.8)	0.0000001

Abbreviations: Se—sensitivity; Sp—specificity; AST—aspartate aminotransferase; IVIG—intravenous immune globulin; MAS—macrophage activation syndrome.

**Table 3:** Regression model results of predictors that allowed us to discriminate MAS in MIS-C patients.

Parameters	β	SE	*p*-Value
Intercept	0.0066	0.021	0.756
Platelets ≤ 114 × 10^9^/L	0.69	0.068	0.0000001
Ferritin > 469 ng/mL	0.33	0.062	0.000001

## Data Availability

The datasets generated during and/or analyzed during the current study are available from the corresponding author upon reasonable request.
